# Acceptability of Yosa, an mHealth App for Between-Session Therapy Support Among Patients and Therapists: Cross-Sectional Survey Study

**DOI:** 10.2196/86214

**Published:** 2026-07-16

**Authors:** Andrew Williams, Rebecca Crochiere

**Affiliations:** 1 Department of Psychology, Williams College, 18 Hoxsey St, Williamstown, MA, 01267, United States, 1 5083699939

**Keywords:** mobile health app, mHealth, digital health, therapy, cognitive behavioral therapy, homework, mental health, technology acceptance model

## Abstract

**Background:**

Completion of homework, defined as therapeutic activities assigned between sessions to reinforce skills and promote behavior change, is strongly linked to therapy outcomes. Yet, homework compliance remains low, potentially due to outdated delivery methods such as paper or email. Mobile health technologies may improve engagement by digitizing therapy tasks and tracking progress. Yosa is a mobile health app designed to facilitate homework delivery and enhance engagement between sessions for patients in therapy.

**Objective:**

The primary aim of this study was to evaluate the perceived acceptability of Yosa among licensed therapists and individuals currently receiving therapy. A secondary aim was to examine whether key Technology Acceptance Model (TAM) constructs predicted attitudes toward and intention to use Yosa. Qualitative feedback was also collected to inform iterative development and future deployment.

**Methods:**

Two cross-sectional surveys were conducted: study 1 with licensed therapists (N=45) and study 2 with current therapy patients (N=96). Participants viewed video demonstrations of Yosa, learned about Yosa’s features, and rated the app on TAM constructs, including perceived usefulness, perceived ease of use, perceived risk, attitude toward, and intention to use Yosa, using 0‐100 scales. For most constructs, higher scores reflected more favorable evaluations, whereas lower perceived risk scores reflected more favorable evaluations. Descriptive statistics and 95% CIs were generated for each construct in both samples, with scores interpreted relative to the neutral midpoint (50). Multiple regression analyses were conducted to examine predictors of attitude and intention to use. Qualitative feedback from the surveys was analyzed thematically.

**Results:**

Therapists and patients reported generally favorable perceptions of Yosa across TAM domains. Among therapists and patients, ratings of the perceived usefulness of the homework feature, therapy journal, and overall app; perceived ease of use; attitudes toward Yosa; and intention to use were all above the midpoint. Perceived risk scores were mild to moderate in patients and moderate in therapists, respectively. Regression analyses indicated that perceived usefulness was a positive predictor of both attitude toward and intention to use Yosa across therapists and patients, while perceived risk was negatively associated with these outcomes in several models. Qualitative themes included requests for additional features, usability enhancements, and data privacy concerns.

**Conclusions:**

Therapists and patients reported generally favorable perceptions of Yosa after reviewing descriptions and video demonstrations of the platform, particularly in terms of usefulness and ease of use, supporting favorable perceptions of its potential acceptability as a digital tool for between-session therapy support. Qualitative feedback informed refinements aimed at reducing perceived risks and enhancing the intention to use.

## Introduction

### Background

Therapy can be effective in helping individuals manage and achieve remission from mental health disorders and issues [1]. However, only a proportion of patients achieve such desired outcomes in therapy. For example, only 30% to 60% of patients in therapy achieve remission or significant symptom reduction in common psychological disorders, such as depression and eating disorders [2,3]. Thus, changes need to be made to the therapeutic process to improve patient outcomes.

One area in which therapeutic processes could be improved is the patients’ ability to apply the skills learned in therapy to their everyday lives [4], which is often achieved via homework assigned to the patient. Homework refers to assignments that patients complete between sessions to advance treatment goals, such as gaining insight into one’s maladaptive thoughts or behaviors or practicing skills to manage symptoms [5]. Homework assignments are considered essential in most cognitive behavioral therapies and are frequently used in therapeutic practice [5].

Homework assignments can take several forms, including psychoeducational homework, self-monitoring, and behavioral practice [6,7]. Psychoeducational homework may include assigned reading to learn about the symptoms of a person’s psychological disorder, its causes, and other pertinent information related to treatment. Self-monitoring involves tracking one’s cognitions, emotions, and behaviors, which helps the therapist understand a patient’s patterns beyond what is observed in therapy [5] and increases the patient’s awareness of their own internal experiences (thoughts and emotions) and behaviors [8]. Finally, behavioral practice refers to homework that encourages clients to try a new behavioral skill, such as a coping strategy (eg, journaling or meditation) or a behavioral experiment (eg, exposure to an anxiety-provoking situation), fostering skill development and cognitive and behavioral change [6,7].

Homework compliance is significantly correlated with positive patient outcomes across a number of psychological disorders, including mood disorders [9], anxiety disorders [10], trauma or stressor-related disorders [11], and substance use disorders [4]. Meta-analyses have demonstrated greater effectiveness among treatments that include homework compared to those without homework, producing a moderate improvement in outcomes [12].

Despite its clear positive impact, engagement with homework outside of therapy sessions among patients remains low. Homework compliance rates in adults receiving therapy are estimated to be about 50% [13]. The existing literature describes several provider-cited barriers to homework compliance, including difficulty engaging patients, discouragement due to low engagement, uncertainty about what to assign, lack of time, and challenges in individualizing homework [14]. Barriers cited by patients include a lack of support, instruction, interactivity, and reward for completing homework [13,14].

These barriers may be linked to limitations with current delivery methods, specifically the fact that homework is often administered via paper, oral instructions, and/or email. Paper worksheets can be easily lost, may have associations with school and high effort [6], and are not feasible in teletherapy. Homework assigned via oral instruction may lack clarity and can be easily forgotten. Furthermore, homework delivered via paper, oral instruction, and email lacks interactivity, which is a barrier to compliance with homework for patients [14].

Mobile health (mHealth) apps provide several advantages over traditional methods of delivering homework. A mobile app centralizes assignments, making them accessible anytime, and reducing the risk of lost materials. Given the ubiquity of smartphones, which an estimated 91% of the US population uses and carries almost constantly [15], mobile apps are almost always at a user’s fingertips. Additionally, mobile apps may enhance engagement with homework through interactive features, gamification, reminders, notifications, a stimulating user interface, user customization, and self-monitoring capabilities. Their data-tracking features may enable easier progress monitoring and pattern identification. For example, therapists could see a patient’s symptom ratings, substance use, medication adherence, and activity in one place, making it easy to spot patterns in the data, for example, how spikes in stress coincide with alcohol use.

To date, all known therapy homework apps are designed for specific therapy approaches (eg, cognitive behavioral therapy) or disorders (ie, posttraumatic stress disorder) with a library of premade assignments, limiting customization and flexibility. A shortcoming of this type of app is that therapists often use multiple therapy approaches and treat diverse patients with different presenting problems. Furthermore, a rigid library of premade assignments reduces therapists’ ability to introduce new worksheets, use worksheets outside of the existing library, or tailor worksheets to individual patients. Thus, to our knowledge, existing apps fail to allow for this customization and personalization around homework assignments, highlighting the need for a flexible app that allows therapists to tailor assignments to their caseloads.

In addition, despite the availability of thousands of mental health apps, few have undergone rigorous scientific testing. As of 2018, over 325,000 mHealth apps existed—a 10-fold increase from 2014—but only 131 acceptability studies were published [16]. Without acceptability testing among key stakeholders, there is a risk of disconnect between what developers believe will be effective and what therapists and patients find useful. Such testing also provides an opportunity to gather real-world feedback, enabling iterative improvements that better align app features with user needs and ultimately support higher adoption and long-term effectiveness [17].

The challenges inherent in existing modes of delivering homework inspired the development of Yosa, an app designed to improve homework delivery and completion in therapy, with the goal of ultimately improving homework compliance, patient engagement, and outcomes. Acceptability testing is necessary to ensure Yosa meets the needs of the target population and is engaging, easy to use, and low-risk. Thus, evaluations of Yosa from key stakeholders allow us to shape the development of the app so that it offers a viable solution to the homework challenges described above.

### Study Objectives

**Figure 1.**
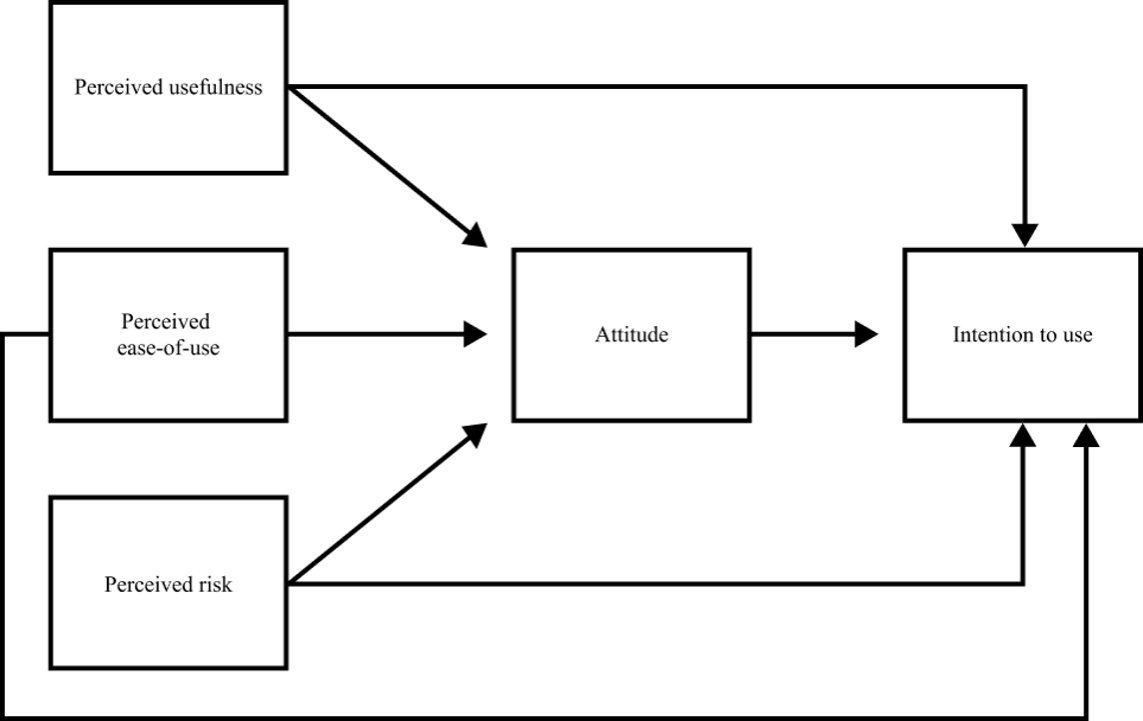
Research model based on the Technology Acceptance Model.

The primary aim of this study is to test Yosa’s acceptability among key stakeholders to inform its potential integration into therapeutic practices, with study 1 surveying licensed therapists and study 2 surveying therapy patients. The Technology Acceptance Model (TAM), the most commonly-used framework for assessing mHealth acceptance [18], was used as a guiding theoretical framework. Specifically, this study addressed the following research questions: (1) How do licensed therapists and current therapy patients rate the acceptability of Yosa across key dimensions of the TAM, including perceived usefulness, perceived ease of use, perceived risk, attitudes, and intention to use Yosa? (2) Which perceptions of Yosa (eg, perceived usefulness) predict attitudes toward and intention to use Yosa? We hypothesized that patients and therapists would rate Yosa as acceptable on the TAM’s scales measuring perceived usefulness, perceived ease of use, attitude, intention to use, and perceived risk. Furthermore, we hypothesized that perceived usefulness and perceived ease of use would positively predict attitude and intention to use Yosa, and perceived risk would negatively predict attitude and intention to use Yosa among both therapists and patients. Although TAM specifies directional relationships among constructs, this study examined these associations using regression-based analyses rather than formally testing the full structural model. Although Yosa is designed to improve engagement between sessions, we were unable to assess actual engagement in this preliminary, survey-based study; thus, engagement was not directly assessed in this study. The research model is shown in Figure 1.

## Methods

### Study 1

#### Participants

Licensed therapists (N=45) with advanced degrees were included in the final analytic sample. Initially, 52 therapists completed the survey; 7 were excluded because they did not complete at least 25% of the study measures. For this preliminary study, convenience sampling was used, and participants were recruited primarily from the Williams College alumni directory and the authors’ professional networks. Participants were required to meet the following inclusion criteria: (1) currently licensed as a therapist, (2) residing in the United States, (3) able to understand and provide informed consent, and (4) proficient in speaking, reading, and writing English. To maximize privacy, identifying information was separated from participant responses. Ten meals were donated to Feeding America for each completed participant response.

#### Procedure

Therapists provided informed consent and then completed questions related to demographics and their therapy practice, including the type and setting of therapy (remote vs in-person), the average number of patients on their caseload, whether they assign homework in therapy, and, if so, the frequency and estimated compliance of homework in their practice, as well as homework delivery methods. Next, therapists read descriptions of Yosa and viewed app video demonstrations of its features, which illustrated various Yosa functionalities. See Multimedia Appendix 1 for screenshots and feature descriptions of the Yosa features included in this study. Participants were not able to directly interact with Yosa, as the app was not yet fully developed at the time of the study. Accordingly, videos were used to gather formative feedback to inform refinements prior to release. The first video was titled *Homework on Yosa* and demonstrated how Yosa allows therapists to upload and send homework assignments via the app, and how patients can view and complete the assignments directly in the app. The next video was titled *Therapy Journal on Yosa* and demonstrated how Yosa allows patients to track therapy sessions, think through presession topics, and generate postsession reflections. The final video was titled *Total Yosa Features* and illustrated how patients can use Yosa to track their thoughts, emotions, and behaviors (eg, medication, sleep, and substance use), journal, generate a safety plan, and access crisis resources.

Each demonstration was followed by a modified version of the TAM perceived usefulness measure, which assessed the usefulness of that specific feature. Therapists then completed the TAM measure of perceived ease of use with all features in mind. Next, therapists learned about the Yosa team’s plan for implementing data security and answered a set of perceived risk items. Afterward, therapists completed measures of their attitude toward and intention to use Yosa as a whole. Finally, therapists were asked if they had any thoughts regarding how to improve Yosa or feedback they would like to share with the founder, allowing for qualitative insights.

#### Ethical Considerations

All study procedures were approved by the Williams College Institutional Review Board via expedited review on February 28, 2024, prior to the study’s commencement. All study procedures adhered to institutional and ethical guidelines for research involving human participants. All participants provided written informed consent, and each participant retained the right to make an informed, voluntary decision to participate in this study. Identifying information was collected solely for the consent process and was not linked to survey data, which were deidentified before analysis. Participants received compensation in the form of charitable donations (10 meals donated to Feeding America) for each completed survey response.

#### Measures

##### TAM

Consistent with prior research, each item measuring perceived usefulness, perceived ease of use, perceived risk, attitude toward, and intention to use the technology (Yosa) was scored on a 100-point scale from “Strongly disagree” (scored as 0) to “Strongly agree” (scored as 100), with a score of 50 representing “Neither agree nor disagree”; all items were averaged equally [19]. Consistent with standard applications of the TAM, constructs were treated as continuous variables. Mean scores and 95% CIs were used to characterize participant responses. Scores were interpreted relative to the scale midpoint (50), which served as a reference point rather than a theoretically derived acceptability threshold. Higher scores on positively valenced constructs (perceived usefulness, perceived ease of use, attitude, and intention to use) reflect more favorable perceptions, and lower scores on perceived risk reflect more favorable perceptions. Because this study used adapted TAM constructs from prior research, internal consistency reliability was assessed using Cronbach α. Exploratory factor analyses were conducted separately for each multi-item TAM construct to evaluate whether items loaded onto their expected dimensions. Analyses supported unidimensionality within constructs, with items demonstrating strong loadings onto single factors across both samples. A broader exploratory factor analysis including all TAM items was explored but produced unstable estimation results, likely reflecting substantial overlap among theoretically related constructs and the modest sample size. All survey items are provided in Multimedia Appendix 2.

##### Perceived Usefulness

This scale assesses the degree to which a particular technology is perceived as useful and has demonstrated excellent internal consistency (Cronbach α=0.97) [20]. Consistent with existing research, participants rated 6 modified TAM items tailored to the specific Yosa feature category being evaluated [21]. For example, participants were asked to indicate how strongly they agreed with statements such as, “Using Yosa would make homework delivery easier.”

##### Perceived Ease of Use

This scale evaluates how easy a particular technology is to use and has demonstrated strong reliability in previous research (Cronbach α=0.93) [20]. Participants rated Yosa on 6 unmodified TAM items assessing ease of use [20]. For example, participants were asked to indicate how strongly they agreed with statements such as, “Learning to operate Yosa would be easy for me.”

##### Perceived Risk

This scale measures concerns related to privacy and security. The 5 items were based on prior studies that draw inspiration from the TAM model (Cronbach α=0.97) [22]. Participants were asked to indicate how strongly they agreed with statements such as, “Using Yosa would result in unauthorized access to sensitive information.”

##### Attitude

This scale measures overall perspectives and opinions of a particular technology, including both positive and negative sentiments about the technology (Guttman lower bound=0.85) [20,23]. For example, participants were asked how strongly they agreed with statements such as, “Using Yosa is a good idea.”

##### Intention to Use

This scale measures willingness and plans to use a particular technology (Cronbach α=0.87) [24]. Participants were asked how strongly they agreed with statements like, “Assuming that I have access to Yosa, I intend to use it.”

Internal consistency was high across measures, with Cronbach α values of 0.95 for homework perceived usefulness, 0.95 for therapy journal perceived usefulness, 0.97 for other features perceived usefulness, 0.92 for perceived ease of use, 0.88 for perceived risk, 0.85 for attitude, and 0.98 for intention to use.

### Study 2

#### Participants

Participants (N=100) completed the patient survey via Prolific [25]. Participants were required to meet the following inclusion criteria: (1) be a current patient receiving mental health therapy; (2) reside in the United States; (3) have the ability to understand and provide informed consent; (4) be proficient in speaking, reading, and writing English; and (5) own or have access to a smartphone (to ensure they could hypothetically use Yosa’s mobile app). No identifying information was collected, and participants received US $4 as compensation after study completion, a rate above Prolific’s guidelines for sufficient compensation for the survey length.

#### Procedure

All study procedures were approved by the Williams College Institutional Review Board prior to the study’s commencement. The patient survey was almost identical to the therapist survey described in study 1, aside from some small modifications detailed below. After providing informed consent, patients completed demographic questions, then read descriptions and viewed video demonstrations of the same features on Yosa, which were slightly modified to better reflect the patient (vs the therapist) perspective. Each demonstration was again followed by items assessing perceived usefulness. After completing the final perceived usefulness measure, patients similarly completed the TAM measure of perceived ease of use, learned about the Yosa team’s plan regarding data security, and completed measures of perceived risk, attitude toward, and intention to use Yosa as a whole. To ensure attentive participation, 2 attention check questions were included within the survey. Participants who failed both attention checks were not compensated or included in the data analysis.

#### Measures

The same TAM measures of perceived usefulness, perceived ease of use, perceived risk, attitude toward, and intention to use Yosa, as described in study 1, were used with minor wording modifications to cater to a patient’s perspective rather than a therapist’s. In study 2, internal consistency was high across measures, with Cronbach α values of 0.94 for homework perceived usefulness, 0.95 for therapy journal perceived usefulness, 0.95 for other features perceived usefulness, 0.94 for perceived ease of use, 0.97 for perceived risk, and 0.85 for attitude.

#### Data Analysis

R Studio (R Foundation for Statistical Computing) was used for statistical analysis. In study 1, survey responses in the Qualtrics survey were not required, resulting in missing data. Given that high item nonresponse threatens statistical power and introduces bias [26], participants were excluded if they answered fewer than 25% of key measure questions, which was considered insufficient for reliable estimates. For measures with 6 items (perceived usefulness and perceived ease-of-use), if more than 1 item was missing, the participant’s scores were excluded. For measures with four or fewer items (perceived risk, attitude, and intention to use), the participant’s scores were dropped if there were any missing items. In study 2, participant responses were excluded if they completed the survey in too little time (<9.5 min) to have watched the videos (7 min total), read the material, and answered the questions. Overall, exclusions across studies primarily reflected incomplete survey responses, missing key measure data, failed attention checks, ineligibility, or unusually rapid completion times.

Before primary analyses, three covariates were identified as being significantly related to key outcomes. First, homework perceived usefulness scores significantly differed between therapists who assign homework and those who do not (*t*_43_=−2.42; *P*=.02; do not assign: mean 56.94, SD 18.77; assign: mean 70.46, SD 21.28). Therefore, data analysis of the perceived usefulness of the Homework feature included only therapists who assign homework. Second, therapists’ intention-to-use Yosa scores significantly differed by highest education level (*t*_39_=−2.24; *P*=.03), with those holding master’s degrees (mean 83.17, SD 25.55) reporting higher intentions to use than those with a doctorate (mean 60.83, SD 33.17). As a result, education level was entered as a covariate in the therapist model predicting intention to use. Third, patients’ intention to use scores significantly differed by therapy delivery type (*F*_2,89_=3.82; *P*=.03). Post hoc Tukey tests indicated that patients receiving in-person therapy (mean 70.00, SD 24.53) reported significantly lower intention to use compared with those receiving remote therapy (mean 83.33, SD 17.96; *P*=.04) or a combination of remote and in-person therapy (mean 82.86, SD 18.74; *P*=.04). This variable was accordingly controlled for in patient regressions with intention to use as the dependent variable.

Average TAM construct scores were generated for both therapist and patient samples. Means and 95% CIs were used to characterize responses across constructs, with scores interpreted relative to the neutral midpoint (50). Perceived usefulness was assessed separately for the homework feature, therapy journal, and overall app (total), but regression analyses within the TAM framework used the overall perceived usefulness (total) measure to capture participants’ holistic evaluation of Yosa’s usefulness when predicting attitude and intention to use.

Open-ended responses were analyzed by 2 independent coders using a structured, blinded thematic analysis protocol. Each coder first read all comments to familiarize themselves with the content, then independently assigned 1 or more codes to each comment to capture its core idea. After this first round of coding, the analysts met to compare codes and jointly develop a shared codebook. Using only the agreed-upon codebook, they then independently recoded all comments again. Interrater reliability was assessed as percent agreement, with a priori agreement set at 80% or more. Agreement did not fall below this threshold. Finally, the team collapsed related codes into broader themes and selected representative quotations. The full procedure was repeated separately for therapist and patient datasets. Correlation matrices of key study constructs are provided in Multimedia Appendix 3. This study is reported in accordance with the STROBE (Strengthening the Reporting of Observational Studies in Epidemiology) guidelines (Checklist 1).

## Results

### Study 1

#### Descriptive Statistics

Seven participants were excluded from the analysis because they completed less than 25% of the key measure questions, bringing the final sample to 45 participants. Multimedia Appendix 4 provides summary statistics on the demographic information of participants, as well as several therapy-related variables. The mean age was 45.3 (SD 12.47) years. The number of years practicing therapy ranged from 1 to 40 years, with an average of 12.93 (SD 11.7) years. The majority of participants reported their assigned sex as female (n=33, 73%) and identified as White or European American (n=35, 78%). All participants held either a master’s degree or a doctorate. The majority of participants practiced cognitive behavioral therapy (n=33, 73%), practiced multiple types of therapy (n=40, 89%), practiced under a private practice (n=23, 51%), saw adult patients (n=40, 89%), and practiced therapy both remotely and in person (n=34, 76%), with just one participant solely practicing in-person. Notably, the majority of participants reported assigning homework to patients in therapy (n=41, 91%).

Multimedia Appendix 5 provides descriptive statistics on homework-related variables for therapists who reported assigning homework (n=41). Therapists who assigned homework did so for 61% (n=25) of their patients on average, and 39% (n=16) assigned homework during every session. Furthermore, therapists who assigned homework estimated, on average, that just 51% of the homework they assigned was completed by patients. Homework delivery methods included paper, email, and oral instruction, with only 15% (n=6) of participants citing other methods.

#### Key Measures

Descriptive and summary statistics of key measures for therapists are shown in Table 1. Overall, therapists reported favorable perceptions of Yosa across most domains, with mean scores generally above the neutral midpoint (50). Ratings of perceived usefulness for the therapy journal (mean 77.85, SD 14.37; 95% CI 73.48-82.22) and overall app features (mean 77.97, SD 19.78; 95% CI 71.89-84.06), perceived ease-of-use (mean 83.00, SD 11.48; 95% CI 79.55-86.45), and attitude toward Yosa (mean 79.37, SD 14.40; 95% CI 74.88-83.85) were notably high. Ratings of perceived usefulness of the Homework feature (mean 70.46, SD 21.28; 95% CI 63.74-77.18) and intention to use (mean 61.08, SD 28.85; 95% CI 51.97-70.18) were comparatively lower, though still above the midpoint. Perceived risk (mean 29.84, SD 19.78; 95% CI 22.58-37.09) was relatively low, indicating limited concern overall. A flow diagram detailing participant inclusion and missing data is presented in Multimedia Appendix 6.

**Table 1.** Descriptive statistics of acceptability measures^a^.

Measure	Sample, n	Min-max	Mean (SD; 95% CI)
Therapists			
Perceived usefulness (homework)	41	5.56-100	70.46 (21.28; 63.74-77.18)
Perceived usefulness (therapy journal)	44	50.00-100	77.85 (14.37; 73.48-82.22)
Perceived usefulness (total)	43	16.67-100	77.97 (19.78; 71.89-84.06)
Perceived ease-of-use	45	56.67-100	83.00 (11.48; 79.55-86.45)
Perceived risk	31	8.33-87.50	29.84 (19.78; 22.58-37.09)
Attitude	42	50.00-100	79.37 (14.40; 74.88-83.85)
Intention to use	41	0.00-100	61.08 (28.85; 51.97-70.18)
Patients			
Perceived usefulness (homework)	96	25.00-100	76.87 (16.75; 73.34-80.40)
Perceived usefulness (therapy journal)	96	16.67-100	75.89 (18.16; 72.21-79.57)
Perceived usefulness (total)	96	16.67-100	77.10 (18.56; 73.34-80.86)
Perceived ease-of-use	96	50.00-100	85.58 (12.90; 82.96-88.19)
Perceived risk	96	0.00-100	33.62 (25.12; 28.44-38.79)
Attitude	96	16.67-100	78.42 (18.09; 74.74-82.11)
Intention to use	96	16.67-100	79.53 (20.83; 75.21-83.84)

a Therapists who do not assign homework rated the perceived usefulness of homework significantly lower than those who did assign homework. Thus, the perceived usefulness of homework for therapists only includes therapists who do assign homework. Differences in sample sizes across therapist measures reflect optional responses and occasional missing data.

#### Regression Results

Panel A of Table 2 presents regression results with perceived usefulness (total), perceived ease-of-use, and perceived risk predicting attitude toward or intention to use Yosa, respectively. For attitude, the model explained 80% of the variance (*R²*=0.80; adjusted *R²*=0.77; *P*=.01). Perceived usefulness (total) was a positive predictor of attitude (*b*=0.55, SE=0.08; *P*=.01), and perceived risk was a negative predictor (*b*=−0.29, SE=0.08; *P*=.01), whereas perceived ease-of-use was not a significant predictor (*b*=0.16, SE=0.14; *P*=.25). For intention to use, the model also explained 80% of the variance (*R²*=.80; adjusted *R²*=0.77; *P*=.01). Perceived usefulness (total) was the only significant predictor of intention to use (*b*=1.14, SE=0.13; *P*=.01); perceived ease-of-use and perceived risk were not significant predictors (*b*=0.29, SE=0.26, *P*=.27; *b*=−0.20, SE=0.15, *P*=.19, respectively).

**Table 2.** Regression analyses of Technology Acceptance Model (TAM) predictors of attitude and intention to use Yosa^a^.

	*b*^b^ (SE, 95% CI)	*P* value
Panel A: therapists		
Attitude toward Yosa (*R*^2^=0.80)		
Intercept	32.43 (12.53, 6.51 to 58.35)	*.*02^c^
Perceived usefulness (total)	0.55 (0.08, 0.39 to 0.71)	.01^c^
Perceived ease-of-use	0.16 (0.14, −0.12 to 0.44)	.25
Perceived risk	−0.29 (0.08, −0.45 to −0.13)	*.*01^c^
Intention to use Yosa (*R*^2^=0.80)		
Intercept	−44.34 (23.83, −93.53 to 4.85)	.08
Perceived usefulness (total)	1.14 (0.13, 0.87 to 1.40)	.01^c^
Perceived ease-of-use	0.29 (0.26, −0.25 to 0.83)	.27
Perceived risk	−0.20 (0.15, −0.50 to 0.10)	.19
Panel B: patients		
Attitude toward Yosa (*R*^2^=0.69)		
Intercept	33.19 (9.23, 14.83 to 51.55)	.01^c^
Perceived usefulness (total)	0.61 (0.06, 0.48 to 0.73)	.01^c^
Perceived ease-of-use	0.06 (0.09, −0.11 to 0.24)	.45
Perceived risk	−0.21 (0.05, −0.31 to −0.11)	.01^c^
Intention to use Yosa (*R*^2^=0.66)		
Intercept	40.01 (12.22, 15.72 to 64.30)	.01^c^
Perceived usefulness (total)	0.81 (0.08, 0.65 to 0.97)	*.*01^c^
Perceived ease-of-use	−0.21 (0.11, −0.43 to 0.01)	.06
Perceived risk	−0.19 (0.07, −0.32 to −0.06)	*.*01^c^

a Predictors were entered simultaneously in multiple regression models.

b “*b*” refers to the unstandardized regression coefficient.

c Significant *P* value.

### Study 2

#### Descriptive Statistics

After excluding those who completed the survey unusually quickly or failed attention checks, 100 participants completed the survey. Four participants were not current therapy patients, bringing the final sample to 96 participants. Multimedia Appendix 4 provides summary statistics on the demographic information of participants. The mean age was 37 (SD 11.18) years. The majority of participants were women (n=60, 61%), identified as White or European American (n=55, 57%), and had obtained a bachelor’s degree (n=40, 42%). The majority of participants received cognitive behavioral therapy (n=60, 63%), attended a private practice (n=77, 80%), and had used a mental health app before (n=55, 57%). Receiving therapy in-person or remotely was approximately evenly split. Notably, the vast majority of participants were assigned homework in therapy (n=89, 93%).

Multimedia Appendix 7 provides descriptive statistics on homework-related variables for patients who were assigned homework (89/96). Among patients who were assigned homework, frequency of assigned homework varied, with the most common frequency being every few sessions (n=40/89, 44.9%). Furthermore, patients who were assigned homework estimated, on average, that they completed 72% of assignments. Homework delivery methods included paper, email, and oral instruction, with just 5 (6%) participants reporting other methods.

#### Key Measures

Descriptive and summary statistics of key measures for patients are shown in Table 1. Overall, patients reported favorable perceptions of Yosa across all domains, with mean scores well above the neutral midpoint (50). Ratings of perceived usefulness of the homework feature (mean 76.87, SD 16.75; 95% CI 73.34-80.40), therapy journal (mean 75.89, SD 18.16; 95% CI 72.21-79.57), and overall app (mean 77.10, SD 18.56; 95% CI 73.34-80.86) were high. Perceived ease-of-use (mean 85.58, SD 12.90; 95% CI 82.96-88.19), attitude toward Yosa (mean 78.42, SD 18.09; 95% CI 74.74-82.11), and intention to use (mean 79.53, SD 20.83; 95% CI 75.21-83.84) were also notably high. Perceived risk (mean 33.62, SD 25.12; 95% CI 28.44-38.79) was relatively higher compared to other domains, though still below the midpoint.

#### Regression Results

Panel B of Table 2 presents the patient regressions with perceived usefulness (total), perceived ease-of-use, and perceived risk predicting attitude or intention to use Yosa, respectively. For attitude, the model explained 69% of the variance (*R²*=0.69; adjusted *R²*=0.68). Perceived usefulness (total) was a positive predictor of attitude (*b*=0.61, SE=0.06; *P*=.01), and perceived risk was a negative predictor (*b*=−0.21, SE=0.05; *P*=.01), whereas perceived ease-of-use was not a significant predictor (*b*=0.06, SE=0.09; *P*=.45). For intention to use, the model explained 66% of the variance (*R²*=0.66; adjusted *R²*=0.65). Perceived usefulness (total) was a positive predictor of intention to use (*b*=0.81, SE=0.08; *P*=.01), and perceived risk was a negative predictor (*b*=−0.19, SE=0.07; *P*=.01); perceived ease-of-use had a trend-level relation with intention to use (*b*=−0.21, SE=0.11; *P*=.06).

### Qualitative Data

Multimedia Appendix 8 presents the thematic analysis results among therapists and patients. Thirty-five therapists provided comments, which were categorized into 6 themes: additional features (n=14), general homework efficacy problems (n=12), data privacy concerns (n=7), enhanced patient monitoring (n=4), positive feedback (n=4), and enhanced user experience (n=2). Sixty-five patients provided comments, which were categorized into seven themes: positive feedback (n=24), enhanced user experience (n=13), additional features (n=13), general homework efficacy problems (n=6), data privacy concerns (n=6), refining existing features (n=5), and help resources (n=2). Because individual comments sometimes addressed multiple ideas and could be assigned to multiple themes, theme counts exceed the number of unique comments. Representative sample quotes for each theme are provided in Multimedia Appendix 8.

## Discussion

### Principal Findings

This study’s exploration of Yosa’s acceptability highlights a potential advancement in integrating mHealth technology within therapeutic practices, particularly offering a promising path for improving homework delivery and completion. Patients reported favorable perceptions of Yosa across all domains, while therapists also rated several features positively. Specifically, both groups rated the perceived usefulness of the therapy journal and overall app, ease of use, and attitudes toward Yosa highly, with mean scores well above the neutral midpoint. However, therapists reported comparatively lower ratings of the perceived usefulness of the homework feature and intention to use, whereas patients reported more favorable ratings across these domains. Perceived risk was higher relative to other constructs for both groups, indicating an area for improvement.

Therapists’ lower intention to use ratings compared to patients may reflect the habits and routines developed through years of delivering therapy in established ways. Patients, in contrast, may be less anchored to a single model of how therapy should look, making them more open to adopting tools like Yosa. Resistance to adopting new technology is common, particularly when transitioning from conventional to modernized methods requires additional effort [27]. Reducing workflow frictions and conducting future studies demonstrating evidence-based benefits of Yosa over current therapeutic methods may be crucial in bolstering Yosa’s credibility and increasing therapists’ interest in adopting the technology. Financial incentives through remote therapeutic monitoring billing codes may also be an important part of adoption not captured in this study [28].

Inconsistent with hypotheses, perceived risk was relatively higher for both groups, suggesting a need to address security concerns. Improving transparency around patient health information (PHI) protections, such as obtaining Health Insurance Portability and Accountability Act (HIPAA) compliance, may help alleviate these concerns [29]. Additionally, peer influence plays a role in technology adoption [30], and therapists may serve as key “opinion leaders” for patients [31]. Since patients in this study did not receive therapist endorsements of Yosa, real-world adoption may differ if therapists specifically recommend the app to a patient during treatment.

Notably, nearly nine out of ten therapists reported drawing on two or more therapeutic approaches (eg, cognitive behavioral therapy and acceptance and commitment therapy), underscoring the value of Yosa’s flexibility. Because Yosa is not restricted to any one therapy type, it may provide an advantage over many existing homework apps that are confined to a single treatment model. By supporting diverse assignments, Yosa may be well-positioned to meet the real-world needs of practitioners who blend techniques to suit individual patients or adapt their approach based on presenting problems. Although prior work has explored tracking and self-monitoring features in digital health tools [6,8], Yosa extends these functions by integrating homework delivery, therapy journaling, therapist feedback, and between-session support within a flexible psychotherapy-oriented platform.

The regression analyses showed that the regression models based on TAM constructs explained a large proportion of the variability in both stakeholders’ attitudes toward Yosa and their willingness to use it, suggesting these TAM constructs were strongly associated with attitudes toward and intention to use Yosa in this sample. Though a formal analysis was not conducted to compare the strength of predictors in the TAM regression models, perceived usefulness (total) seemed to have a particularly strong relationship with attitude and intention to use for both patients and therapists. Although these results do not necessarily support causality, the TAM posits that perceived usefulness is a key determinant of intention to use [20]. As such, increasing Yosa’s perceived usefulness may be especially important to increase adoption. Thus, the Yosa team should prioritize developing its utility, and the qualitative feedback gathered in this study offers clear guidance on where to focus.

Interestingly, perceived ease of use was not a significant predictor of attitude, and only perceived usefulness emerged as a consistent predictor of intention to use. One possible explanation is the high overall ratings of ease of use, which may have limited variability and reduced its predictive power. Additionally, participants evaluated Yosa based on video demonstrations rather than direct interaction, which may have made it more difficult to meaningfully assess usability. It is also possible that in early-stage evaluations, participants prioritize whether a tool appears beneficial over considerations of usability or risk, which may become more salient during real-world use.

Thematic analysis of qualitative feedback from therapists and patients revealed insights into Yosa’s strengths and areas for improvement. Many content suggestions were provided, including the addition of more trackers, validated measures like the Patient Health Questionnaire–9 [32] and Generalized Anxiety Disorder–7 [33], and notifications or reminders. Usability issues regarding the PDF format of homework assignments were mentioned multiple times, with suggestions for improvement, such as making homework assignments be fillable and shareable with therapists. Integration with electronic health record systems was also suggested multiple times by therapists. Additionally, privacy concerns were prevalent, especially among therapists. The app’s esthetics and user interface generally received positive feedback, with the suggestion to incorporate more animations and illustrations.

Beyond identifying specific feature requests, the qualitative findings also suggest several potential mechanisms underlying participants’ favorable perceptions of Yosa. Many responses point to the importance of convenience and reducing barriers to completing therapeutic activities, particularly by making tools more accessible and integrated into daily life. Comments emphasizing simplicity, usability, and replacing static formats (eg, PDFs) with interactive features suggest that reducing friction may be a key factor in perceived usefulness. Although prior work on digital mental health tools has often emphasized engagement, symptom tracking, or gamification, the present findings suggest that reducing practical barriers and friction in homework completion may also be central to perceived acceptability and willingness to use digital therapy tools. This may represent an important consideration for future digital mental health intervention design. Together, these findings suggest that Yosa’s appeal may stem from its ability to make therapy tasks more manageable, accessible, and integrated into everyday routines.

Overall, this study provides preliminary evidence of the acceptability and potential demand for Yosa as a digital health tool for therapists and patients, warranting its development into a fully functional app. Given the well-established connection between homework completion and better therapy outcomes, and the fact that patients complete only about half of the homework assigned to them [13], Yosa’s potential ability to keep patients engaged between sessions and enhance homework completion could meaningfully improve patient outcomes.

### Strengths and Limitations

There are many strengths of this study. Engagement with diverse stakeholders is considered essential for successful mental health interventions [34]. Thus, perhaps the most important strength of the study is the input from both therapists and patients. By gathering data from each, this study offers a more comprehensive perspective on the acceptability and potential adoption of Yosa. This allows for a nuanced understanding of the app’s potential impact across treatment, distinguishing it from other technology evaluations, which typically assess only one stakeholder group [16]. Additionally, integrating a comments section for qualitative feedback enhanced the design by complementing numerical measures of Yosa’s acceptability with nuanced reflections on Yosa’s strengths, weaknesses, and areas for improvement. We strengthened this design by recruiting 2 independent coders to conduct the qualitative analysis, helping preserve objectivity and reduce bias. Furthermore, to improve transparency and reduce potential bias related to the founder’s involvement, survey responses were anonymized, participants were informed that responses would remain confidential, and the second author independently supervised and reviewed the study design, analyses, and interpretation of findings. Finally, the use of Prolific for data collection in study 2, which is praised for its high quality [25], allowed for a fairly diverse sample in age, race, and gender, enhancing the external validity of findings. Attention checks and the exclusion of participants with unusually fast completions also increased data integrity in study 2.

Despite numerous strengths, the limitations of the study are also acknowledged. Study 1 relied on convenience sampling through professional networks, which may limit external validity and increase the risk of response biases, including demand characteristics and social desirability effects. To help mitigate these concerns, participants were informed that responses would be anonymized, only the research team would have access to the data, participation was voluntary, and withdrawal was permitted at any time without penalty. Nonetheless, favorable response bias remains possible. Furthermore, the samples of therapists in study 1 and patients in study 2 were predominantly White, limiting generalizability to underrepresented groups. Additionally, the sample was skewed toward individuals receiving care in private practice settings and slightly toward those with prior experience using mental health apps, which may limit generalizability. Participants receiving care in private practice may also have relatively greater socioeconomic resources and greater access to, or familiarity with, smartphone technology than other therapy patients, which may have contributed to more favorable perceptions of Yosa. Similarly, participants with prior experience using digital mental health tools may have been more comfortable adopting new technologies, potentially inflating acceptability ratings. Next, although this study was guided by the TAM, it did not formally test the full structural pathways specified by TAM using structural equation modeling. Given the modest sample size, particularly for the therapist sample, the analyses focused on regression-based associations among constructs. Future research with larger samples should test the full TAM structural model more directly.

Additionally, participants evaluated Yosa based on video demonstrations rather than direct interaction with the app. As a result, findings reflect perceived acceptability and hypothetical intention to use rather than actual behavior, and real-world usability may differ once users interact with the fully functional application. Accordingly, findings should be interpreted as perceptions of anticipated usefulness, ease of use, risk, attitudes, and intention to use based on descriptions and demonstrations of Yosa rather than direct user experience with the application. Future studies should also examine how perceptions of usefulness, ease of use, and risk change following sustained real-world interaction with the application, as these perceptions may evolve during actual clinical use. Another limitation of study 1 was nonmandatory responses, leading to missing data, potential nonresponse bias, and reduced statistical power, which affected estimate reliability. Study 2 corrected this issue by requiring responses for all questions. In study 2, while attention and duration checks helped ensure that the data included in the study represented participants who were attentive and engaged with the study material, there was no way to verify if respondents fully watched the video demonstrations. If participants skipped or partially viewed the videos, their responses might not accurately reflect informed perspectives on Yosa. Next, as comments were optional, the qualitative data are susceptible to nonresponse bias. Additionally, the sample sizes, particularly for the therapist sample (N=45), may limit statistical power, especially for regression analyses. Although we were able to detect statistically significant effects in these models, the smaller sample sizes may have reduced our ability to detect more modest associations and increased the risk of Type II error. Additionally, the relatively high R² values observed in the regression models should be interpreted cautiously. Because all TAM constructs were assessed using self-report measures within the same cross-sectional survey, shared method variance and conceptual overlap among constructs may have inflated associations between variables. Finally, priming can significantly affect risk assessments. Drawing attention to potential risks of Yosa may have induced feelings of perceived risk. If this was the case, results would be biased toward increased perception of risk.

### Future Research Directions

This study lays the foundation for several future research directions. Since conducting this study, the Yosa team has developed a fully functional mobile app for patients and a web portal for therapists, incorporating iterative improvements based on user feedback from this research (see Multimedia Appendix 9 and Multimedia Appendix 10 for demonstrations of the updated applications). Enhancements include gamification, animations, fillable and customized worksheets, meditation, breathing exercises, a library of therapeutic worksheets (eg, thought records), improved usability, customization, and enhanced monitoring features for therapists. Notably, both the app and web portal have been developed in alignment with HIPAA standards.

Future research should involve a clinical trial with a treatment group of patients in therapy using Yosa and a control group of patients in therapy not using Yosa to evaluate Yosa’s impact on engagement between sessions and therapeutic outcomes. Promising results may increase user confidence, trust, and adoption of this tool. As Yosa continues to evolve, ongoing user feedback and iterative improvement will be critical to guide its development toward greater acceptability and, ultimately, effectiveness in mental health care.

## Supplementary material

10.2196/86214 Multimedia Appendix 1 Screenshots and feature descriptions of the Yosa mobile health (mHealth) application used in this study.

10.2196/86214 Multimedia Appendix 2 Survey items used to assess Technology Acceptance Model constructs in study 1 (therapists) and study 2 (patients).

10.2196/86214 Multimedia Appendix 3 Correlation matrix for primary study constructs in study 1 (therapists) and study 2 (patients).

10.2196/86214 Multimedia Appendix 4 Participant demographic characteristics in study 1 (therapists) and study 2 (patients).

10.2196/86214 Multimedia Appendix 5 Descriptive statistics of therapists that assign homework.

10.2196/86214 Multimedia Appendix 6 Participant flow diagram and exclusion criteria for study 1 and study 2.

10.2196/86214 Multimedia Appendix 7 Descriptive statistics of patients assigned homework.

10.2196/86214 Multimedia Appendix 8 Qualitative themes and representative quotes from study 1 and study 2.

10.2196/86214 Multimedia Appendix 9 Video demonstration of the current version of the Yosa mobile application, reflecting updates made following therapist and patient feedback gathered in the feasibility and acceptability study reported in this manuscript.

10.2196/86214 Multimedia Appendix 10 Video demonstration of the current version of the Yosa therapist web portal, reflecting updates made following therapist and patient feedback gathered in the feasibility and acceptability study reported in this manuscript.

10.2196/86214 Checklist 1 STROBE checklist.

## Abbreviations

HIPAA: Health Insurance Portability and Accountability Act
mHealth: mobile health
PHI: Protected Health Information
STROBE: Strengthening the Reporting of Observational Studies in Epidemiology
TAM: Technology Acceptance Model
