# Supplementary material for: Acceptability of Yosa, an mHealth App for Between-Session Therapy Support Among Patients and Therapists: Cross-Sectional Survey Study
Source: JMIR Form Res. 2026 Jul 16;10:e86214. doi: 10.2196/86214 (PMC13375209; doi:10.2196/86214)

**Multimedia Appendix 1. Screenshots and Feature Descriptions of the Yosa Application**

The first feature group, “Homework on Yosa”, reimagines the delivery and completion of traditional homework worksheets, akin to those familiar in grade school, into a digital format. While currently commonly delivered through email PDF format or paper, on Yosa, therapists can upload a worksheet digitally and send it to a patient. Patients, in turn, can view and complete their assignments directly on the app. An example of what this would look like from a patient’s perspective is shown below, which was shown to participants in the survey video demonstration. In this example, this user’s therapist had assigned the worksheet “Challenging Negative Thoughts”, which upon clicking, can be viewed and responded to by toggling between the “PDF” and “Response” buttons.


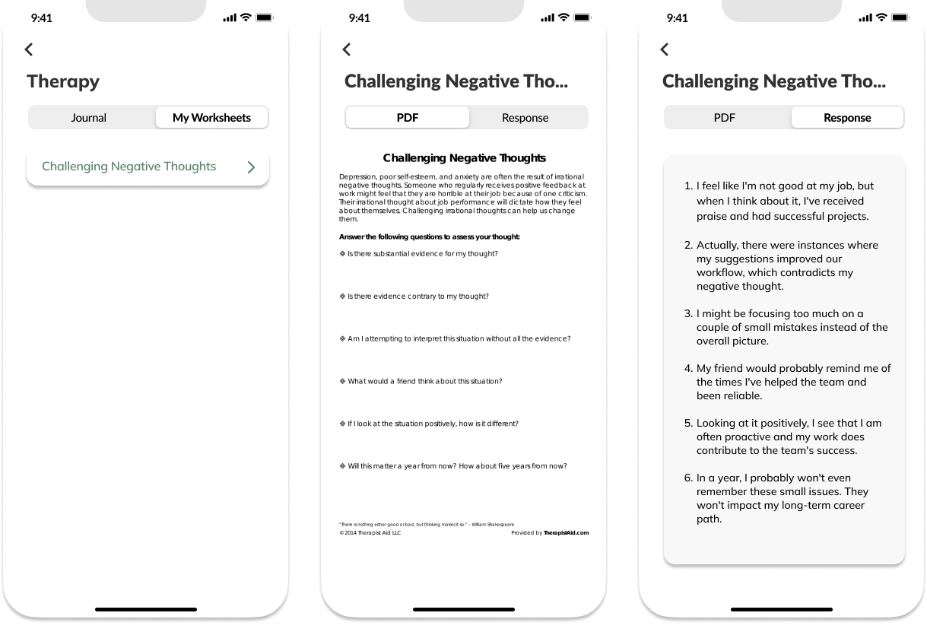


The next feature shown to therapists, the “Therapy Journal on Yosa”, allows patients to track therapy sessions, aiming to increase patient engagement, and help patients become active participants in therapy by helping think through session expectations, pre-session topics, and post-session reflections. The image below shows mock-ups of the Therapy Journal on Yosa that were included in the survey demonstration. The leftward screen shows the journal’s dashboard, where users can view past entries and create a new entry. The middle screen shows the “Before” portion of the Therapy Journal, which asks users a series of questions in preparation of a given therapy session. The rightward screen shows the “After” portion of the Therapy Journal, asking users to reflect on their therapy session.


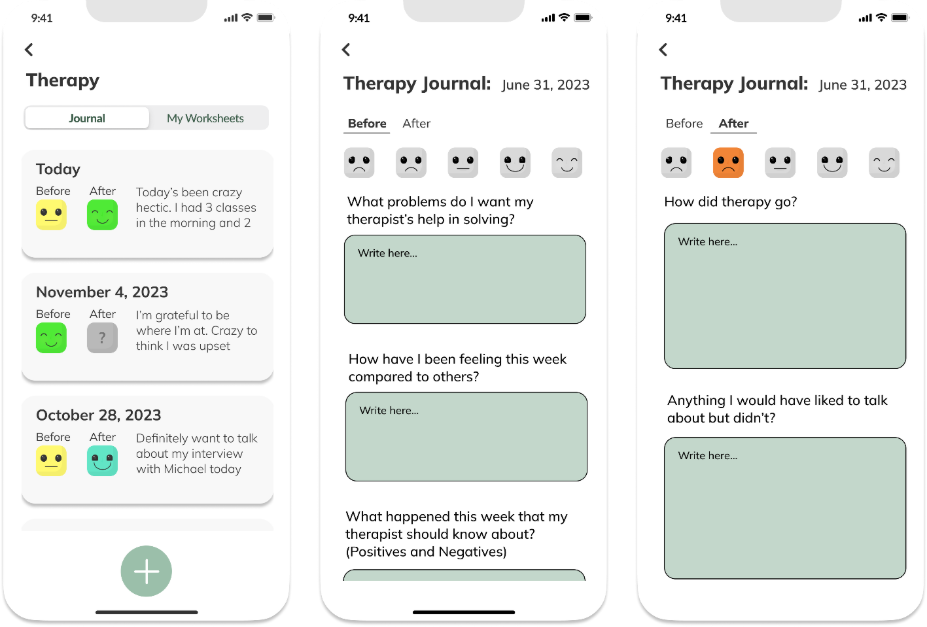


The final feature group shown to therapists was “Other Yosa Features”. The image below shows the Yosa home screen and all its features, many of which may be considered homework in between therapy sessions, including various trackers (mood, medication, sleep, substances), journaling, a safety plan, and help resources. Three specific “Other Yosa Features” that were shown to participants during the study demonstration are the Mood Tracker, Safety Plan, and Help resources, shown below


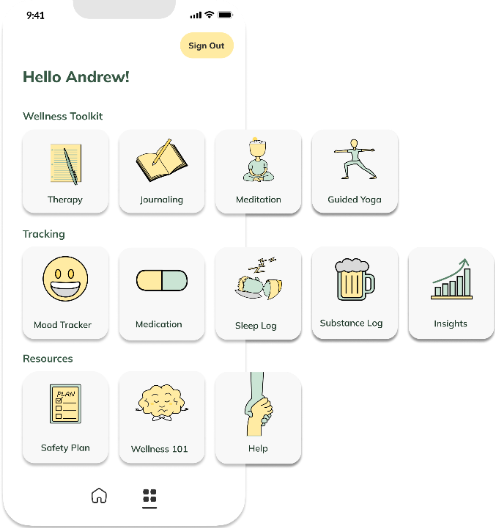


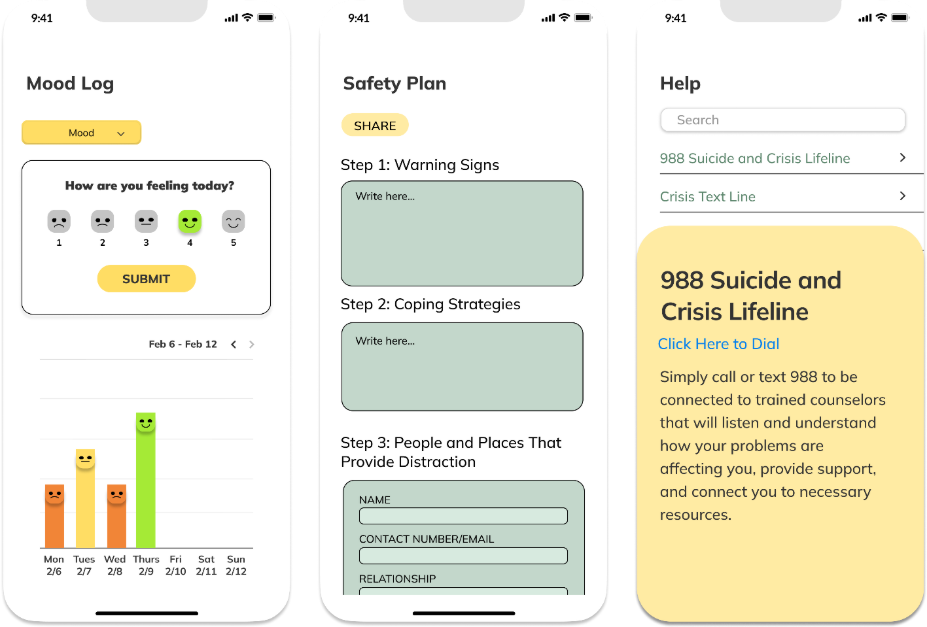

Supplement: Multimedia Appendix 1 [file formative-v10-e86214-s001.docx]
