# Supplementary material for: Acceptability of Yosa, an mHealth App for Between-Session Therapy Support Among Patients and Therapists: Cross-Sectional Survey Study
Source: JMIR Form Res. 2026 Jul 16;10:e86214. doi: 10.2196/86214 (PMC13375209; doi:10.2196/86214)
Supplement: Multimedia Appendix 2 [file formative-v10-e86214-s002.docx]

**Multimedia Appendix 2. Survey Items**

The following are items from study measures.

## *Study 1*

**Perceived Usefulness**

*Scale: ”Strongly disagree” to “Strongly agree” (1-7)*

*Homework on Yosa*

PU1: Using Yosa's homework feature would support critical aspects of my job.

PU2: Using Yosa's homework feature would improve my therapy practice.

PU3: Using Yosa would increase my patients' homework compliance.

PU4: Using Yosa would make homework delivery easier.

PU5: Using Yosa's homework feature would benefit my patients.

PU6: I would find Yosa's homework feature useful in my job.

*Therapy Journal on Yosa*

PU1: My patients' use of the therapy journal would improve engagement in therapy.

PU2: Using Yosa would improve my therapy practice.

PU3: My patients' use of the therapy journal would improve patient outcomes.

PU4: My patients' use of the therapy journal would help track progress in therapy.

PU5: Using Yosa's therapy journal would benefit my patients.

PU6: My patients would find Yosa's therapy journal useful.

*Total Yosa Features*

PU1: Using Yosa would improve my therapy practice.

PU2: Using Yosa would improve patient outcomes.

PU3: Using Yosa would support critical aspects of my job.

PU4: I would find Yosa useful in my job.

PU5: My patients would find Yosa useful.

PU6: Using Yosa would benefit myself or my patients.

**Perceived Ease of Use**

*Scale: ”Strongly disagree” to “Strongly agree” (1-7)*

PEOU1: Learning to operate Yosa would be easy for me.

PEOU2: I would find it easy to get Yosa to do what I want it to do.

PEOU3: My interaction with Yosa would be clear and understandable.

PEOU4: It would be easy for me to remember how to perform tasks using Yosa.

PEOU5: It would be easy for me to become skillful at using Yosa.

PEOU6: I would find Yosa easy to use.

**Perceived Risk**

*Scale: ”Strongly disagree” to “Strongly agree” (1-7)*

PR1: Using this in my therapy practice would be risky.

PR2: My signing up for and using this would lead to a loss of privacy for me or my patients.

PR3: Using this would result in unauthorized access to sensitive information.

PR4: This is dangerous to use.

PR5: Using this exposes you to overall risk.

**Attitude**

*Scale: ”Strongly disagree” to “Strongly agree” (1-7)*

A1: Using Yosa is a good idea.

A2: Using Yosa is a wise idea.

A3: I like the idea of using Yosa.

A4: Using Yosa would be pleasant.

**Intention to Use**

*Scale: ”Strongly disagree” to “Strongly agree” (1-7)*

IU1: As soon as Yosa becomes available, I plan to use it in my therapy practice.

IU2: Assuming that I have access to Yosa, I intend to use it in my therapy practice.

IU3: Assuming Yosa is available to use, I aim to use it in my therapy practice within the next six (6) months.

IU4: Assuming Yosa is available to use, I anticipate using it in my therapy practice five (5) years from now.

## *Study 2*

**Perceived Usefulness**

*Scale: ”Strongly disagree” to “Strongly agree” (1-7)*

*Homework on Yosa*

PU1: Using Yosa's homework feature would support critical aspects of my mental health treatment.

PU2: Using Yosa's homework feature would improve my mental health treatment.

PU3: Using Yosa would increase my homework compliance.

PU4: Using Yosa would make homework delivery easier.

PU5: Using Yosa's homework feature would benefit me.

PU6: I would find Yosa's homework feature useful in my mental health treatment.

*Therapy Journal on Yosa*

PU1: Using the therapy journal would improve my engagement in therapy.

Attention Check 1: This is an attention check. You must select "Agree" in this row.

PU2: Using the therapy journal would improve my mental health treatment.

PU3: My use of the therapy journal would improve my mental health.

PU4: My use of the therapy journal would help track progress in therapy.

PU5: Using Yosa's therapy journal would benefit me.

PU6: I would find Yosa's therapy journal useful.

*Total Yosa Features*

PU1: Using Yosa would improve my mental health treatment.

PU2: Using Yosa would improve my mental health.

PU3: Using Yosa would support critical aspects of my mental health treatment.

PU4: I would find Yosa useful in my mental health treatment.

PU5: I would find Yosa useful.

PU6: Using Yosa would benefit myself or me.

**Perceived Ease of Use**

*Scale: ”Strongly disagree” to “Strongly agree” (1-7)*

PEOU1: Learning to operate Yosa would be easy for me.

PEOU2: I would find it easy to get Yosa to do what I want it to do.

PEOU3: My interaction with Yosa would be clear and understandable.

Attention Check 2: This is an attention check. You must select "Disagree" in this row.

PEOU4: It would be easy for me to remember how to perform tasks using Yosa.

PEOU5: It would be easy for me to become skillful at using Yosa.

PEOU6: I would find Yosa easy to use.

**Perceived Risk**

*Scale: ”Strongly disagree” to “Strongly agree” (1-7)*

PR1: Using Yosa would be risky.

PR2: My signing up for and using Yosa would lead to a loss of privacy for me.

PR3: Using Yosa would result in unauthorized access to sensitive information.

PR4: Yosa is dangerous to use.

**Attitude**

*Scale: ”Strongly disagree” to “Strongly agree” (1-7)*

A1: Using Yosa is a good idea.

A2: I like the idea of using Yosa.

**Intention to Use**

*Scale: ”Strongly disagree” to “Strongly agree” (1-7)*

IU1: Assuming my therapist offers the opportunity to use Yosa, and it’s available to me at no cost, I would intend to use it in my mental health treatment.
