# Supplementary material for: Acceptability of Yosa, an mHealth App for Between-Session Therapy Support Among Patients and Therapists: Cross-Sectional Survey Study
Source: JMIR Form Res. 2026 Jul 16;10:e86214. doi: 10.2196/86214 (PMC13375209; doi:10.2196/86214)
Supplement: Multimedia Appendix 3 [file formative-v10-e86214-s003.docx]

**Multimedia Appendix 3. Correlation Matrices**

| *Correlation Matrix for Primary Study Constructs Among Therapists* | | | | | | | |
| --- | --- | --- | --- | --- | --- | --- | --- |
|  | 1 | 2 | 3 | 4 | 5 | 6 | 7 |
| 1. Perceived usefulness (homework) | - |  |  |  |  |  |  |
| 2. Perceived usefulness (therapy journal) | 0.72** | - |  |  |  |  |  |
| 3. Perceived usefulness (total) | 0.77** | 0.77** | - |  |  |  |  |
| 4. Perceived ease-of-use | 0.36* | 0.54** | 0.15 | - |  |  |  |
| 5. Perceived risk | -0.09 | -0.21 | -0.14 | -0.01 | - |  |  |
| 6. Attitude | 0.68** | 0.72** | 0.78** | 0.27 | -0.47* | - |  |
| 7. Intention to use | 0.75** | 0.8** | 0.85** | 0.32* | -0.30 | 0.76** | - |
| *Correlation Matrix for Primary Study Constructs Among Patients* | | | | | | | |
|  | 1 | 2 | 3 | 4 | 5 | 6 | 7 |
| 1. Perceived usefulness (homework) | - |  |  |  |  |  |  |
| 2. Perceived usefulness (therapy journal) | 0.69** | - |  |  |  |  |  |
| 3. Perceived usefulness (total) | 0.77** | 0.85** | - |  |  |  |  |
| 4. Perceived ease-of-use | 0.27** | 0.23* | 0.24* | - |  |  |  |
| 5. Perceived risk | -0.47** | -0.42** | -0.49** | -0.31** | - |  |  |
| 6. Attitude | 0.60** | 0.68** | 0.78** | 0.31** | -0.65** | - |  |
| 7. Intention to use | 0.71** | 0.71** | 0.78** | 0.08 | -0.51** | 0.72** | - |
| * p < .05; ** p < .01 |  |  |  |  |  |  |  |
