# Supplementary material for: Acceptability of Yosa, an mHealth App for Between-Session Therapy Support Among Patients and Therapists: Cross-Sectional Survey Study
Source: JMIR Form Res. 2026 Jul 16;10:e86214. doi: 10.2196/86214 (PMC13375209; doi:10.2196/86214)
Supplement: Multimedia Appendix 4 [file formative-v10-e86214-s004.docx]

**Multimedia Appendix 5. Participant Demographic Characteristics**

| *Descriptive Statistics (Therapists)* |  | |  |  |  |  |
| --- | --- | --- | --- | --- | --- | --- |
|  | | *n* | *min* | *max* | *mean* | *SD* |
| Age | | 44 | 29 | 70 | 45.3 | 12.47 |
| Years Practicing | | 45 | 1 | 40 | 12.93 | 11.67 |
| Caseload | | 42 | 2 | 220 | 43 | 42.29 |
|  | | *n* | % |  |  |  |
| **Sex** | |  |  |  |  |  |
| Female | | 33 | 73% |  |  |  |
| Male | | 10 | 22% |  |  |  |
| Non-binary | | 2 | 4% |  |  |  |
| **Race/Ethnicity** | |  |  |  |  |  |
| White or European American | | 35 | 78% |  |  |  |
| Multiracial | | 6 | 13% |  |  |  |
| Black or African American | | 2 | 4% |  |  |  |
| Asian or Asian American | | 1 | 2% |  |  |  |
| Hispanic/Latino/Latina/Latinx | | 1 | 2% |  |  |  |
| **Highest Education Level** | |  |  |  |  |  |
| Master's Degree | | 34 | 76% |  |  |  |
| Doctorate | | 11 | 24% |  |  |  |
| **Therapy Types Practiced†** | |  |  |  |  |  |
| Cognitive Behavioral Therapy (CBT) | | 33 | 73% |  |  |  |
| Mindfulness-Based Cognitive Therapy (MBCT) | | 19 | 42% |  |  |  |
| Psychodynamic Therapy | | 18 | 40% |  |  |  |
| Trauma-Focused Therapy | | 17 | 38% |  |  |  |
| Family, Couples, or Group Therapy | | 16 | 36% |  |  |  |
| Acceptance and Commitment Therapy (ACT) | | 15 | 33% |  |  |  |
| Humanistic Therapy | | 12 | 27% |  |  |  |
| Dialectical Behavioral Therapy (DBT) | | 11 | 24% |  |  |  |
| Eye Movement Desensitization and Reprocessing (EMDR) | | 7 | 16% |  |  |  |
| Play Therapy | | 5 | 11% |  |  |  |
| Somatic Therapy | | 4 | 9% |  |  |  |
| Other | | 3 | 7% |  |  |  |
| **Therapy Delivery Method** | |  |  |  |  |  |
| Both Remote and In-Person | | 34 | 76% |  |  |  |
| Remote | | 10 | 22% |  |  |  |
| In-Person | | 1 | 2% |  |  |  |
| **Therapy Setting†** | |  |  |  |  |  |
| Private Practice | | 23 | 51% |  |  |  |
| Hospital | | 9 | 20% |  |  |  |
| Military and Veteran Services | | 6 | 13% |  |  |  |
| Schools and Educational Institutions | | 6 | 13% |  |  |  |
| Community Based Center / Program | | 5 | 11% |  |  |  |
| Other | | 4 | 9% |  |  |  |
| Nonprofit Organization | | 3 | 7% |  |  |  |
| **Patient Age Group(s)†** | |  |  |  |  |  |
| Adults (25-64 years) | | 40 | 89% |  |  |  |
| Young Adults (18-24 years) | | 36 | 80% |  |  |  |
| Seniors (65+ years) | | 28 | 62% |  |  |  |
| Adolescents (13-17 years) | | 14 | 31% |  |  |  |
| Children (0-12 years) | | 6 | 13% |  |  |  |
| **Assigns Homework** | |  |  |  |  |  |
| Yes | | 41 | 91% |  |  |  |
| No | | 4 | 9% |  |  |  |
| *Note. For respondents that provided a range for caseload (e.g., 20-40), the average was calculated. † For Therapy Types Practiced, Therapy Setting, and Patient Age Group, where respondents could select multiple options, each row displays the number of respondents that selected that option. Thus, percentages do not equate to 100% for these variables.* | | | | | | |

| *Descriptive Statistics (Patients)* |  |  |  |  |  |
| --- | --- | --- | --- | --- | --- |
|  | *n* | *min* | *max* | *mean* | *SD* |
| Age (years) | 96 | 20 | 63 | 37 | 11.18 |
|  | *n* | % |  |  |  |
| **Gender** |  |  |  |  |  |
| Woman | 60 | 63% |  |  |  |
| Man | 28 | 29% |  |  |  |
| Non-binary / third gender | 5 | 5% |  |  |  |
| Other | 2 | 2% |  |  |  |
| Prefer not to say | 1 | 1% |  |  |  |
| **Race/Ethnicity** |  |  |  |  |  |
| White or European American | 55 | 57% |  |  |  |
| Black or African American | 16 | 17% |  |  |  |
| Multiracial | 11 | 11% |  |  |  |
| Hispanic/Latino/Latina/Latinx | 8 | 8% |  |  |  |
| Asian or Asian American | 2 | 2% |  |  |  |
| Middle Eastern origin | 2 | 2% |  |  |  |
| Native American or Alaska Native | 2 | 2% |  |  |  |
| **Highest Education Level** |  |  |  |  |  |
| Bachelor's degree | 40 | 42% |  |  |  |
| Some college | 36 | 38% |  |  |  |
| High school degree (or equivalent) | 10 | 10% |  |  |  |
| Master's degree | 8 | 8% |  |  |  |
| Doctorate | 2 | 2% |  |  |  |
| **Therapy Types Received†** |  |  |  |  |  |
| Cognitive Behavioral Therapy (CBT) | 60 | 63% |  |  |  |
| Trauma-Focused Therapy | 32 | 33% |  |  |  |
| Other | 14 | 15% |  |  |  |
| Unsure | 13 | 14% |  |  |  |
| Mindfulness-Based Cognitive Therapy (MBCT) | 11 | 11% |  |  |  |
| Dialectical Behavioral Therapy (DBT) | 9 | 9% |  |  |  |
| Family, Couples, or Group Therapy | 8 | 8% |  |  |  |
| Eye Movement Desensitization and Reprocessing (EMDR) | 7 | 7% |  |  |  |
| Acceptance and Commitment Therapy (ACT) | 6 | 6% |  |  |  |
| Humanistic Therapy | 5 | 5% |  |  |  |
| Psychodynamic Therapy | 3 | 3% |  |  |  |
| Somatic Therapy | 3 | 3% |  |  |  |
| **Therapy Delivery Method** |  |  |  |  |  |
| Both remote and in-person | 38 | 40% |  |  |  |
| Remote | 33 | 34% |  |  |  |
| In-person | 25 | 26% |  |  |  |
| **Therapy Setting†** |  |  |  |  |  |
| Private Practice | 77 | 80% |  |  |  |
| Community Based Center / Program | 10 | 10% |  |  |  |
| Other | 8 | 8% |  |  |  |
| Hospital | 6 | 6% |  |  |  |
| Nonprofit Organization | 2 | 2% |  |  |  |
| Military and Veteran Services | 1 | 1% |  |  |  |
| Schools and Educational Institutions | 1 | 1% |  |  |  |
| Rehabilitation Center | 1 | 1% |  |  |  |
| Unsure | 1 | 1% |  |  |  |
| **Ever Used a Mental Health App** |  |  |  |  |  |
| Yes | 55 | 57% |  |  |  |
| No | 41 | 43% |  |  |  |
| **Assigned Homework** |  |  |  |  |  |
| Yes | 89 | 93% |  |  |  |
| No | 7 | 7% |  |  |  |
| *Note. † For Therapy Types Received and Therapy Setting, where respondents could select multiple options, each row displays the number of respondents that selected that option. Thus, percentages do not equate to 100% for these variables.* | | | | | |
