# Supplementary material for: Acceptability of Yosa, an mHealth App for Between-Session Therapy Support Among Patients and Therapists: Cross-Sectional Survey Study
Source: JMIR Form Res. 2026 Jul 16;10:e86214. doi: 10.2196/86214 (PMC13375209; doi:10.2196/86214)
Supplement: Multimedia Appendix 5 [file formative-v10-e86214-s005.docx]

| *Descriptive Statistics of Therapists that Assign Homework* | | | | | |
| --- | --- | --- | --- | --- | --- |
|  | *n* | *min* | *max* | *mean* | *SD* |
| % of Patients Homework is Given To | 37 | 10 | 100 | 61.32 | 29.04 |
| % of Assigned Homework that is Completed | 37 | 10 | 90 | 50.73 | 22.06 |
|  | *n* | % |  |  |  |
| **Homework Assignment Frequency** |  |  |  |  |  |
| Every session | 16 | 39.02% |  |  |  |
| Every other session | 4 | 9.76% |  |  |  |
| Every few sessions | 17 | 41.46% |  |  |  |
| Rarely | 4 | 9.76% |  |  |  |
| **Homework Delivery Method** |  |  |  |  |  |
| Email | 2 | 4.88% |  |  |  |
| Paper | 3 | 7.32% |  |  |  |
| Verbal | 6 | 14.63% |  |  |  |
| Paper, Email | 10 | 24.39% |  |  |  |
| Paper, Verbal | 9 | 21.95% |  |  |  |
| Email, Verbal | 3 | 7.32% |  |  |  |
| Paper, Email, Verbal | 2 | 4.88% |  |  |  |
| Paper, Other | 4 | 9.76% |  |  |  |
| Other | 2 | 4.88% |  |  |  |
|  |  |  |  |  |  |
