# Supplementary material for: Acceptability of Yosa, an mHealth App for Between-Session Therapy Support Among Patients and Therapists: Cross-Sectional Survey Study
Source: JMIR Form Res. 2026 Jul 16;10:e86214. doi: 10.2196/86214 (PMC13375209; doi:10.2196/86214)

**Multimedia Appendix 7. Participant Flow Diagram for Study 1 (Therapists) and Study 2 (Patients)**

Description: Flow diagrams illustrating participant inclusion, exclusion, and final analytic samples for both studies. Sample sizes for each TAM construct are displayed. Sample sizes vary across therapist measures due to missing data from non-required items.

A. Study 1: Therapists


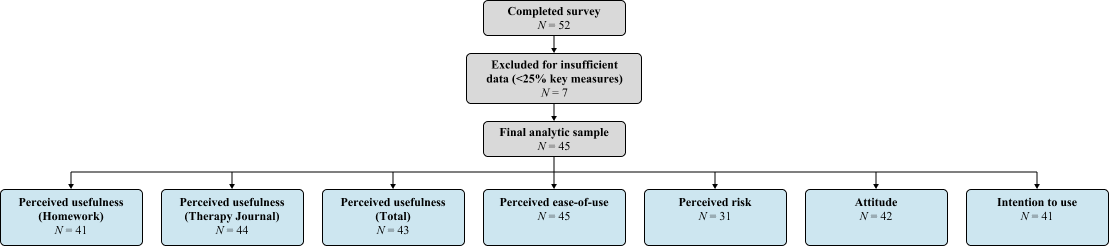


B. Study 2: Patients


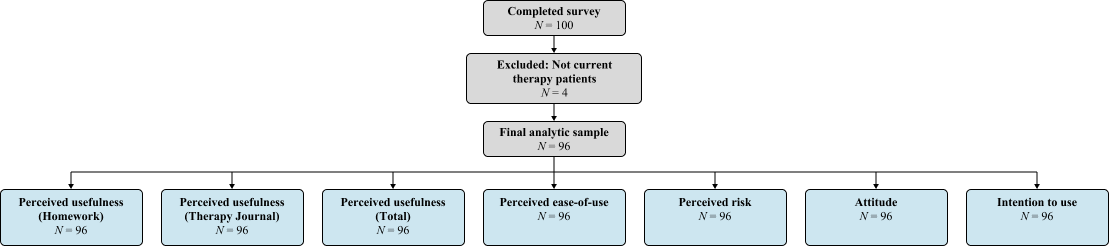

Supplement: Multimedia Appendix 6 [file formative-v10-e86214-s006.docx]
