# Supplementary material for: Acceptability of Yosa, an mHealth App for Between-Session Therapy Support Among Patients and Therapists: Cross-Sectional Survey Study
Source: JMIR Form Res. 2026 Jul 16;10:e86214. doi: 10.2196/86214 (PMC13375209; doi:10.2196/86214)
Supplement: Multimedia Appendix 7 [file formative-v10-e86214-s007.docx]

| *Descriptive Statistics of Patients Assigned Homework* |  |  |  |  |  |
| --- | --- | --- | --- | --- | --- |
|  | *n* | *min* | *max* | *mean* | *SD* |
| % of Assigned Homework that is Completed | 89 | 10 | 100 | 71.99 | 24.99 |
|  | *n* | % |  |  |  |
| **Homework Assignment Frequency** |  |  |  |  |  |
| Every session | 25 | 28.09% |  |  |  |
| Every other session | 12 | 13.48% |  |  |  |
| Every few sessions | 40 | 44.94% |  |  |  |
| Rarely | 12 | 13.48% |  |  |  |
| **Homework Delivery Method** |  |  |  |  |  |
| Email | 6 | 6.74% |  |  |  |
| Paper | 2 | 2.25% |  |  |  |
| Verbal | 40 | 44.94% |  |  |  |
| Paper, Email | 2 | 2.25% |  |  |  |
| Paper, Verbal | 12 | 13.48% |  |  |  |
| Email, Verbal | 14 | 15.73% |  |  |  |
| Paper, Email, Verbal | 8 | 8.99% |  |  |  |
| Verbal, Other | 3 | 3.37% |  |  |  |
| Other | 2 | 2.25% |  |  |  |
