# Supplementary material for: Acceptability of Yosa, an mHealth App for Between-Session Therapy Support Among Patients and Therapists: Cross-Sectional Survey Study
Source: JMIR Form Res. 2026 Jul 16;10:e86214. doi: 10.2196/86214 (PMC13375209; doi:10.2196/86214)
Supplement: Multimedia Appendix 8 [file formative-v10-e86214-s008.docx]

**Multimedia Appendix 9. Qualitative Data Among Therapists and Patients**

| *Qualitative Data Among Therapists* | | |
| --- | --- | --- |
| Theme | *n* | Sample Quote |
| Additional Features | 14 | "I love the simple interface. Working with clients (and coworkers!) during covid taught me that technology can be a huge barrier to care. I think keeping it simple means keeping it accessible. Other trackers could be meals (not specifically what food, just if someone ate a meal or not), water (maybe just measured in # of water bottles drank), physical activity, and energy level. I don't think these are necessary additions, I like what you have now, just other ideas based on what I hear a lot in sessions! As well as other metrics that might be helpful for clients to see in the insights tab (which I absolutely love) e.g. seeing that physical activity and energy both have a positive correlation, while substance use and sleep might have an inverse relationship." |
|  |  |  |
| General Homework Efficacy Problems | 12 | "I do not give homework sheets - that is why I said it would not directly benefit what I do." |
|  |  |  |
| Data Privacy Concerns | 7 | "Love the idea. Only concerns would be related to security/any breach of private information." |
|  |  |  |
| Enhanced Patient Monitoring | 4 | "Having homework assignments be fillable and shareable with the therapist is very important. That way we can both look at the homework together at the same time and discuss it. Ideally, clients would have a "submit" or "share" option on each assignment that lets the therapist see their worksheet/journal/activity exactly as they see it so we can look at it together in session. They could toggle it ‘private' or ‘shared.’" |
|  |  |  |
| Positive Feedback | 4 | “No, looks awesome! Can’t wait to try it!” |
| Enhanced User Experience | 2 | "It might be worthwhile to hire a graphic designer to make the visuals more appealing. The interface in the tutorials looks a bit dated. I also think you'd get the most traction from this in therapy spaces if there were customizable options for prompts. For example, journal questions that the therapist can change." |
|  |  |  |
| *Total* | 43 |  |

| *Qualitative Data Among Patients* | | |
| --- | --- | --- |
| Theme | *n* | Sample Quote |
| Positive Feedback | 24 | "I do not. Yosa seems well-rounded and very well thought out." |
|  |  |  |
| Enhanced User Experience | 13 | "I would make the design more user friendly and easy to access." |
|  |  |  |
| Additional Features | 13 | "I would say instead of a PDF that it would just pop up with an area that you fill in under each question." |
|  |  |  |
|  |  |  |
| General Homework Efficacy Problems | 6 | "I like the idea of this app but finding time to work with it would just be another task for me." |
|  |  |  |
| Data Privacy Concerns | 6 | "I think it’s a great idea for an app. I do know similar things exist out there but this feels like a better "one stop shop" than the other options I've heard people mention. The only thing I worry about is any of the information finding itself on the cloud with the potential for a data breach. When it comes to mental health, privacy is a huge factor for people deciding to seek out or avoid treatment. So I would possibly look into maybe an offline mode (one that doesn’t require a user to have to sign in or permanently log or even upload/save any data)." |
|  |  |  |
| Refine Existing Features | 5 | "One thing I would really caution against is using charity as a reward for completing therapy assignments. Rather than being motivational, that's going to very quickly become stressful and guilt-driven rather than the patient doing the assignments for their own benefit." |
|  |  |  |
| Help Resources | 2 | "Having access to emergency numbers (like 911) in the app can be helpful to someone who is in a mental health situation." |
| *Total* | 69 |  |
