# Supplementary material for: Acceptability of Yosa, an mHealth App for Between-Session Therapy Support Among Patients and Therapists: Cross-Sectional Survey Study
Source: JMIR Form Res. 2026 Jul 16;10:e86214. doi: 10.2196/86214 (PMC13375209; doi:10.2196/86214)
Supplement: Checklist 1 [file formative-v10-e86214-s011.docx]

**STROBE Checklist**

STROBE Statement—checklist of items that should be included in reports of observational studies

|  | Item No. | Recommendation | Page  No. | Relevant text from manuscript |
| --- | --- | --- | --- | --- |
| **Title and abstract** | 1 | (*a*) Indicate the study’s design with a commonly used term in the title or the abstract | Title Page | Acceptability of Yosa, a Mobile Health Application for Between-Session Therapy Support Among Patients and Therapists: A Cross-Sectional Survey Study |
|  |  | (*b*) Provide in the abstract an informative and balanced summary of what was done and what was found | Abstract | Structured abstract including Background, Objective, Methods, Results, and Conclusions summarizing study design, measures, and findings. |
| Introduction | | | |  |
| Background/rationale | 2 | Explain the scientific background and rationale for the investigation being reported | Introduction | Discussion of low homework compliance, limitations of current delivery methods, and potential of mHealth solutions to improve engagement. |
| Objectives | 3 | State specific objectives, including any prespecified hypotheses | Study Objectives | The primary aim of this study is to test Yosa’s acceptability among key stakeholders to inform its potential integration into therapeutic practices... We hypothesized patients and therapists would rate Yosa as acceptable on the TAM’s scales measuring perceived usefulness, perceived ease-of-use, attitude, intention to use, and perceived risk. Further, we hypothesized perceived usefulness and perceived ease-of-use would positively predict attitude and intention to use Yosa, and perceived risk would negatively predict attitude and intention to use Yosa among both therapists and patients. |
| Methods | | | |  |
| Study design | 4 | Present key elements of study design early in the paper | Abstract | Two cross-sectional surveys were conducted… |
| Setting | 5 | Describe the setting, locations, and relevant dates, including periods of recruitment, exposure, follow-up, and data collection | Participants / Procedure | Recruitment via Williams College alumni network (Study 1) and Prolific (Study 2), United States, online survey format. |
| Participants | 6 | (*a*) *Cohort study*—Give the eligibility criteria, and the sources and methods of selection of participants. Describe methods of follow-up  *Case-control study*—Give the eligibility criteria, and the sources and methods of case ascertainment and control selection. Give the rationale for the choice of cases and controls  *Cross-sectional study*—Give the eligibility criteria, and the sources and methods of selection of participants | N/A | N/A |
|  |  | (*b*) *Cohort study*—For matched studies, give matching criteria and number of exposed and unexposed  *Case-control study*—For matched studies, give matching criteria and the number of controls per case | Participants | Eligibility criteria, recruitment sources, and inclusion requirements described for therapists and patients. |
| Variables | 7 | Clearly define all outcomes, exposures, predictors, potential confounders, and effect modifiers. Give diagnostic criteria, if applicable | Measures | Definitions of perceived usefulness, ease-of-use, risk, attitude, and intention to use based on TAM. |
| Data sources/ measurement | 8* | For each variable of interest, give sources of data and details of methods of assessment (measurement). Describe comparability of assessment methods if there is more than one group | Measures | Use of adapted TAM scales; 0–100 response format; Cronbach’s alpha reported; items provided in Multimedia Appendix 2. |
| Bias | 9 | Describe any efforts to address potential sources of bias | Limitations | Convenience sampling, social desirability, demand characteristics, prior app experience, and video-based evaluation limitations discussed. |
| Study size | 10 | Explain how the study size was arrived at | Participants | Sample sizes reported (*N*=45 therapists, *N*=96 patients); no formal power analysis conducted. |

Continued on next page

| Quantitative variables | 11 | Explain how quantitative variables were handled in the analyses. If applicable, describe which groupings were chosen and why | Measures / Data Analysis | Variables treated as continuous; interpreted relative to midpoint (50). |
| --- | --- | --- | --- | --- |
| Statistical methods | 12 | (*a*) Describe all statistical methods, including those used to control for confounding | Data Analysis | Multiple regression models used; covariates included where significant. |
|  |  | (*b*) Describe any methods used to examine subgroups and interactions | Data Analysis | Subgroup differences examined (e.g., homework assignment, education level, therapy type). |
|  |  | (*c*) Explain how missing data were addressed | Data Analysis | Clear rules for excluding participants and handling missing items. |
|  |  | (*d*) *Cohort study*—If applicable, explain how loss to follow-up was addressed  *Case-control study*—If applicable, explain how matching of cases and controls was addressed  *Cross-sectional study*—If applicable, describe analytical methods taking account of sampling strategy | Data Analysis | Convenience sampling acknowledged; no weighting applied. |
|  |  | (*e*) Describe any sensitivity analyses | N/A | N/A |
| Results | | | | |
| Participants | 13* | (a) Report numbers of individuals at each stage of study—eg numbers potentially eligible, examined for eligibility, confirmed eligible, included in the study, completing follow-up, and analysed | Results | Exclusions and final sample sizes reported; flow diagram provided in Multimedia Appendix 7. |
|  |  | (b) Give reasons for non-participation at each stage | Results | Reasons for exclusion (missing data, attention checks, speed). |
|  |  | (c) Consider use of a flow diagram | Results | A flow diagram… is presented in Multimedia Appendix 7. |
| Descriptive data | 14* | (a) Give characteristics of study participants (eg demographic, clinical, social) and information on exposures and potential confounders | Results | Demographics and therapy characteristics (Appendix 5). |
|  |  | (b) Indicate number of participants with missing data for each variable of interest | Results | A flow diagram… is presented in Multimedia Appendix 7. |
|  |  | (c) *Cohort study*—Summarise follow-up time (eg, average and total amount) | N/A | N/A |
| Outcome data | 15* | *Cohort study*—Report numbers of outcome events or summary measures over time | N/A | N/A |
|  |  | *Case-control study—*Report numbers in each exposure category, or summary measures of exposure | N/A | N/A |
|  |  | *Cross-sectional study—*Report numbers of outcome events or summary measures | Results | Means, SDs, and 95% CIs reported for all TAM constructs. |
| Main results | 16 | (*a*) Give unadjusted estimates and, if applicable, confounder-adjusted estimates and their precision (eg, 95% confidence interval). Make clear which confounders were adjusted for and why they were included | Results | Regression coefficients, SEs, and 95% CIs reported; covariates included where relevant. |
|  |  | (*b*) Report category boundaries when continuous variables were categorized | N/A | N/A |
|  |  | (*c*) If relevant, consider translating estimates of relative risk into absolute risk for a meaningful time period | N/A | N/A |

Continued on next page

| Other analyses | 17 | Report other analyses done—eg analyses of subgroups and interactions, and sensitivity analyses | Data Analysis / Results | Subgroup comparisons and thematic analysis conducted. |
| --- | --- | --- | --- | --- |
| Discussion | | | | |
| Key results | 18 | Summarise key results with reference to study objectives | Principal Findings | Summary of favorable perceptions, differences between therapists and patients, and key TAM findings. |
| Limitations | 19 | Discuss limitations of the study, taking into account sources of potential bias or imprecision. Discuss both direction and magnitude of any potential bias | Limitations | Comprehensive discussion of sampling bias, generalizability, power, missing data, and video-based evaluation. |
| Interpretation | 20 | Give a cautious overall interpretation of results considering objectives, limitations, multiplicity of analyses, results from similar studies, and other relevant evidence | Discussion | Interpretation grounded in TAM, prior literature, and qualitative findings. |
| Generalisability | 21 | Discuss the generalisability (external validity) of the study results | Limitations | Discussion of demographic skew, private practice bias, and prior app use. |
| Other information | |  | | |
| Funding | 22 | Give the source of funding and the role of the funders for the present study and, if applicable, for the original study on which the present article is based | Funding Statement | Supported by Williams College… no role in study design… |

*Give information separately for cases and controls in case-control studies and, if applicable, for exposed and unexposed groups in cohort and cross-sectional studies.

**Note:** An Explanation and Elaboration article discusses each checklist item and gives methodological background and published examples of transparent reporting. The STROBE checklist is best used in conjunction with this article (freely available on the Web sites of PLoS Medicine at http://www.plosmedicine.org/, Annals of Internal Medicine at http://www.annals.org/, and Epidemiology at http://www.epidem.com/). Information on the STROBE Initiative is available at www.strobe-statement.org.
